# Gene-level association analysis of systemic sclerosis: A comparison of African-Americans and White populations

**DOI:** 10.1371/journal.pone.0189498

**Published:** 2018-01-02

**Authors:** Olga Y. Gorlova, Yafang Li, Ivan Gorlov, Jun Ying, Wei V. Chen, Shervin Assassi, John D. Reveille, Frank C. Arnett, Xiaodong Zhou, Lara Bossini-Castillo, Elena Lopez-Isac, Marialbert Acosta-Herrera, Peter K. Gregersen, Annette T. Lee, Virginia D. Steen, Barri J. Fessler, Dinesh Khanna, Elena Schiopu, Richard M. Silver, Jerry A. Molitor, Daniel E. Furst, Suzanne Kafaja, Robert W. Simms, Robert A. Lafyatis, Patricia Carreira, Carmen Pilar Simeon, Ivan Castellvi, Emma Beltran, Norberto Ortego, Christopher I. Amos, Javier Martin, Maureen D. Mayes

**Affiliations:** 1 Department of Biomedical Data Science, Geisel School of Medicine, Dartmouth College, Lebanon, NH, United States of America; 2 Department of Internal Medicine, Division of Rheumatology, University of Texas McGovern Medical School, Houston, TX, United States of America; 3 Department of Biostatistics, UT MD Anderson Cancer Center, Houston, TX, United States of America; 4 Wellcome Trust Sanger Institute, Hinxton, United Kingdom; 5 Institute of Parasitology and Biomedicine López-Neyra, IPBLN-CSIC, Granada, Spain; 6 Robert S. Boas Center for Genomics and Human Genetics, Feinstein Institute for Medical Research, Manhasset, NY, United States of America; 7 Division of Rheumatology, Georgetown University Medical Center, Washington, D.C., United States of America; 8 Division of Rheumatology, University of Alabama—Birmingham, Birmingham, AL, United States of America; 9 Division of Rheumatology, University of Michigan, Ann Arbor, MI, United States of America; 10 Division of Rheumatology, Medical University of South Carolina, Charleston, SC, United States of America; 11 Division of Rheumatic and Autoimmune Diseases, University of Minnesota, Minneapolis, MN, United States of America; 12 Division of Rheumatology, University of California—Los Angeles, Los Angeles, CA, United States of America; 13 University of Washington, Seattle, WA, United States of America; 14 University of Florence, Florence, Italy; 15 Division of Rheumatology, Boston University, Boston, MA, United States of America; 16 University of Pittsburgh, Pittsburgh, PA, United States of America; 17 Department of Rheumatology, Hospital Universitario, Madrid, Spain; 18 Department of Internal Medicine, Valle de Hebrón Hospital, Barcelona, Spain; 19 Hospital de la Santa Creu i Sant Pau, Barcelona, Spain; 20 Hospital Universitario y Politécnico La Fe, Valencia, Spain; 21 University Hospital San Cecilio, Granada, Spain; Keio University, JAPAN

## Abstract

Gene-level analysis of ImmunoChip or genome-wide association studies (GWAS) data has not been previously reported for systemic sclerosis (SSc, scleroderma). The objective of this study was to analyze genetic susceptibility loci in SSc at the gene level and to determine if the detected associations were shared in African-American and White populations, using data from ImmunoChip and GWAS genotyping studies. The White sample included 1833 cases and 3466 controls (956 cases and 2741 controls from the US and 877 cases and 725 controls from Spain) and the African American sample, 291 cases and 260 controls. In both Whites and African Americans, we performed a gene-level analysis that integrates association statistics in a gene possibly harboring multiple SNPs with weak effect on disease risk, using Versatile Gene-based Association Study (VEGAS) software. The SNP-level analysis was performed using PLINK v.1.07. We identified 4 novel candidate genes (*STAT1*, *FCGR2C*, *NIPSNAP3B*, and *SCT*) significantly associated and 4 genes (*SERBP1*, *PINX1*, *TMEM175* and *EXOC2*) suggestively associated with SSc in the gene level analysis in White patients. As an exploratory analysis we compared the results on Whites with those from African Americans. Of previously established susceptibility genes identified in Whites, only *TNFAIP3* was significant at the nominal level (p = 6.13x10^-3^) in African Americans in the gene-level analysis of the ImmunoChip data. Among the top suggestive novel genes identified in Whites based on the ImmunoChip data, *FCGR2C* and *PINX1* were only nominally significant in African Americans (p = 0.016 and p = 0.028, respectively), while among the top novel genes identified in the gene-level analysis in African Americans, *UNC5C* (p = 5.57x10^-4^) and *CLEC16A* (p = 0.0463) were also nominally significant in Whites. We also present the gene-level analysis of SSc clinical and autoantibody phenotypes among Whites. Our findings need to be validated by independent studies, particularly due to the limited sample size of African Americans.

## Introduction

Systemic sclerosis (SSc, scleroderma) [MIM 181750] is an autoimmune disease characterized by three key features: (1) fibrosis of skin and internal organs, (2) a vasculopathy, and (3) autoantibody production. It is a multiorgan system disease with considerable phenotypic heterogeneity, resulting in a broad spectrum of disease severity. Several genome-wide, ImmunoChip, and follow-up association studies were conducted to identify SNPs associated with SSc risk [1–8]. All published studies implemented SNP-level analysis meaning that each SNP was analyzed separately and those with genome wide level of statistical significance were deemed risk associated. SNP-level analysis is effective for identification of SNPs with strong individual effects, however, it is underpowered to detect genes carrying multiple SNPs in the same gene of small or medium effect size [9–11]. In the latter case, gene-level analysis can be beneficial because it will detect genes with multiple small effect size SNPs as significant even if these genes do not harbor any individual SNPs significant at the genome-wide level. However, a gene-level analysis has never been applied to SSc.

In this study we performed a gene-level analysis focusing on the data generated by the ImmunoChip platform. We compared results from the gene-level analysis with the results generated by traditional SNP-level analysis. We also performed a gene-level analysis of ImmunoChip and genome-wide association study (GWAS) data on of African-American SSc patients. Although based on a relatively small group of patients, this study represents the first report of genetic analysis of African Americans with SSc. The results of the gene-level analysis of SSc clinical phenotypes (limited SSc (lcSSc) and diffuse SSc (dcSSc)) as well as autoantibody subsets (anti-centromere autoantibodies (ACA) and anti-DNA topoisomerase I (ATA) autoantibodies) among Whites are also presented.

## Materials and methods

The study has been approved by the Institutional Review Boards of the participating US institutions, namely Boston University; Georgetown University; Medical University of South Carolina; University of Alabama, Birmingham; University of California Los Angeles; University of Michigan; University of Minnesota; University of Washington; University of Pittsburgh; University of Texas Health Science Center, Houston, University of Texas MD Anderson Cancer Center, Houston, Geisel School of Medicine, Dartmouth College; and ethics committees of the participating foreign institutions, namely Institute of Parasitology and Biomedicine López-Neyra, IPBLN-CSIC, Granada, Spain; University of Florence, Florence, Italy; Hospital Universitario, Madrid, Spain; Valle de Hebrón Hospital, Barcelona, Spain; Hospital de la Santa Creu i Sant Pau, Barcelona, Spain; Hospital Universitario y Politécnico La Fe, Valencia, Spain; University Hospital San Cecilio, Granada, Spain. All clinical investigation has been conducted according to the principles expressed in the Declaration of Helsinki. Written informed consent has been obtained from the participants.

Race of the study participants was self-reported, and we used principal component analyses to remove race outliers as described below. Details on the White population, genotyping and quality control can be found in our previously published manuscript [7]. The White sample included 956 cases and 2741 controls from the US and 877 cases and 725 controls from Spain, after exclusion of individuals based on quality control (QC) (low call rates, non-European ancestry, or relatedness). There were 1087 (59%) White patients with lcSSc, 574 (31%) with dcSSc, 671 (37%) ACA-positive (ACA+), and 347 (19%) ATA+ patients (not all patients could be classified into two distinct phenotypes or had either ACA or ATA antibodies).

The African American sample, after quality control measures, included 291 cases (56 men and 235 women) and 260 controls (72 men and 188 women). In line with [12], the distribution of clinical phenotypes and autoantibody subsets in the African American patient population was markedly different from that in Whites. There were 82 (28%) patients with lcSSc, 201 (69%) with dcSSc, 21 (7%) ACA+, and 69 (24%) ATA+ among the African American cases (as with Whites, not all patients could be classified into two distinct phenotypes or had either ACA or ATA antibodies two patients were not tested for ACA and four for ATA).

ImmunoChip analysis: Genotyping was done by Illumina Infinium single-nucleotide polymorphism (SNP) microarray–ImmunoChip. Genotype calling was done using the Illumina iScan System and the Genotyping Module (v.1.8.4) of the GenomeStudio Data Analysis software. We applied the following criteria for QC: (1) individuals with call rate <90% were excluded, (2) markers with call rates ≤ 90% were excluded, and (3) markers with allele distributions deviating from Hardy-Weinberg equilibrium (HWE) in controls (p < 1× 10^−5^) were also excluded. A total 126,270 markers (101,692 of them with a MAF > 0.1%) passed QC and were included in the analysis.

The same ImmunoChip platform was used for White and African American populations. The genotyping rate was 0.988 in the African American sample while the genotyping rate was 0.998 among Whites.

GWAS analysis: We also performed a genome-wide genotyping of both African Americans and Whites. African Americans were genotyped on the Illumina Omni2.5 BeadChip that features ~2.5 million markers capturing variants down to MAF 2.5% and covers, in particular, African genetic diversity. The same exact individuals that were successfully genotyped on ImmunoChip were successfully genotyped on this platform. The genotyping rate after QC was 0.993 in African American population. The quality control for African Americans also included principal component analysis as implemented in SNP & Variation Suite v.7 (Golden Helix). The first three principal components were derived for each individual from the African American sample along with HapMap Phase 2 samples as reference populations. Individuals deviating for more than 6 SDs from the African ancestry cluster centroid were discarded from further analysis. We also excluded individuals deviating more than 4 standard deviations from the cluster centroid. Finally, we excluded duplicate and closely related samples (PIHAT ≥ 0.5).

The genome wide genotyping of the White populations has been described previously in Radstake et al (2010) [3]. In brief, Hap550K-BeadChip was used for US Whites and Illumina HumanCNV370K BeadChip in Spanish Whites.

For the SNP-level analysis the association statistics was computed via logistic regression including sex as a covariate for each dataset. For the White samples, meta-analysis combining odds ratios (OR) and standard errors (SE) of individual datasets (US and Spanish, so that the controls in each set were from the same country as cases) was performed by means of the inverse-variance method under the assumption of a fixed effect as implemented in PLINK v.1.07. [13].

For the gene-level analysis we used Versatile Gene-based Association Study (VEGAS) [14]. We used VEGAS because it outperforms similar methods by sensitivity and specificity from simulation studies [15]. VEGAS can be applied to the data generated by any GWAS designs, including family-based GWAS, meta-analyses of GWAS and DNA-pooling-based GWAS. The test uses information from the complete set of markers within a gene. To account for linkage disequilibrium between markers VEGAS uses simulations from the multivariate normal distribution. VEGAS assigns SNPs to autosomal genes according to positions on the UCSC Genome Browser hg18 assembly. In order to capture regulatory regions and SNPs in LD, the gene boundaries are defined as ±50 kb of 5’ and 3’ UTRs. VEGAS assigned SNPs genotyped by ImmunoChip to 11,501 genes. Assuming independence of the gene level tests, the threshold for statistical significance in the analysis of ImmunoChip in Whites was set to be 4.35x10^-6^, and at 2.8x10^-6^ for the analysis of GWAS data [14]. However, since this threshold is likely to be conservative given the overlap between genes, we report findings with p-values <10^−5^. Also, since the sample size for African Americans was limited, for this population we present findings with the p-value below 10^−3^, acknowledging that this is a study limitation and that the analysis is exploratory and is in need for further validation. We use the term “nominal significance” to denote p-values in the range of 0.05 to 10^−3^, interpreting them as weak evidence of association. We excluded HLA region from the analysis because it is universally significant.

We used PathwayStudio [16] to build a pathway of known and novel SSc risk-associated genes. The PathwayStudio uses text mining to identify reported interactions between genes and build a network based on the known interactions.

## Results

### ImmunoChip analysis

Table 1 shows results from the gene-level and SNP-level analyses for 19 non-HLA genes previously shown to be associated with SSc in Whites [1–8, 17, 18]. Out of 19 known SSc genes, all except for *SCHIP1*, *IRF8*, and *CD247* were nominally significant in the gene-level analysis in Whites. *IRF5*, *STAT4*, and *TNPO3* were significant in both the SNP- and gene-level analyses among Whites.

**Table 1:** Results of the gene-level analyses for known genes associated with risk of SSc in Whites and African Americans, based on ImmunoChip.

			Whites					African	Americans		
Gene	Chr	N_SNPs_	P-value	Top SNP		Top SNP P-value	N_SNPs_	P-value	Top SNP		Top SNP P-value
*STAT4*	2	113	***2*.*00E-09***	rs10174238	*	4.65E-13	108	0.105	rs1551443	*	**0.0106**
*TNPO3*	7	102	***3*.*00E-08***	rs17340351	**	2.39E-10	105	0.314	rs1495461	^§^	0.0574
*IRF5*	7	49	***2*.*00E-06***	rs12534421	*	7.42E-10	53	0.211	rs1495461	^‡^	0.0574
*TNIP1*	5	124	**4.90E-05**	rs10463312	*	4.87E-06	156	0.189	rs2277940	^§^	**0.00742**
*IL12RB2*	1	69	**5.40E-05**	rs2201584	*	1.29E-06	84	0.921	rs11209045	**	**0.0418**
*BLK*	8	176	**1.43E-04**	rs2409781	*	2.99E-08	172	0.552	rs11250139	**	**0.0244**
*ITGAM*	16	53	**1.96E-04**	rs1143683	^†^	5.70E-05	67	0.337	rs4561481	**	**0.00771**
*IL12RB1*	19	42	**0.001144**	rs2305743	*	7.23E-05	48	0.215	rs426132	*	0.0733
*TNFAIP3*	6	45	**0.00145**	rs892999	**	0.0103	69	**0.00613**	rs10223636	*	**0.00203**
*IL12A*	3	110	**0.00248**	rs4679867	*	8.79E-05	141	0.741	rs4679867	*	0.0508
*TYK2*	19	60	**0.00363**	rs2304256	^†^	1.61E-04	63	0.758	rs8108709	*	0.1514
*ATG5*	6	54	**0.0076**	rs11758079	**	0.00276	62	0.272	rs4946731	**	**0.0083**
*CSK*	15	8	**0.0129**	rs6495122	^§^	1.96E-05	9	0.787	rs8033381	*	0.299
*JAZF1*	7	96	**0.0325**	rs6971086	*	5.64E-04	112	0.844	rs3823946	*	**0.0122**
*PXK*	3	180	**0.0397**	rs7626140	*	0.00699	187	0.755	rs9870786	*	**0.0177**
*DNASE1L3*	3	84	**0.0464**	rs4681786	**	3.09E-04	93	0.597	rs9843169	**	**0.0177**
*CD247*	1	5	0.0821	rs2056626	*	0.00541	8	0.467	rs10918695	*	0.211
*IRF8*	16	24	0.0986	rs10863202	**	0.0165	28	0.518	rs11642456	**	0.129
*SCHIP1*	3	81	0.174	rs1675497	*	0.00108	77	0.449	rs17753641	*	0.0543

*Intronic SNP

**intergenic SNP

^†^coding SNP

^§^3’ downstream SNP

^‡^5’ upstream SNP

In the gene-level analysis of clinical phenotypes (S1 and S2 Tables) and antibody subsets (S3 and S4 Tables), *STAT4* was significant for lcSSc and ACA+ patients and *TNPO3* in ATA+ patients. Of the 19 genes examined, only *TNFAIP3* was nominally significant in African Americans in the gene-level analysis.

Table 2 shows non-HLA genes with the p-values below 10^−5^ in the gene-level analysis for Whites (excluding those already established in Whites), with the addition of p-values for these genes in African Americans. The genes with the p-values below 4.35x10^-6^ are shown in bold.

**Table 2:** Comparison of novel candidate genes (at p<4.35x10^-6^ indicated in bold) and suggestive genes (4.35x10^-6^<p<10^−5^) detected in Whites to the corresponding statistics in African Americans in the gene level analysis, based on ImmunoChip.

			Whites					African	Americans		
Gene	Chr	N_SNPs_	P-value	Top SNP		Top SNP p-value	N_SNPs_	P-value	Top SNP		Top SNP p-value
**STAT1**	2	60	<1.00E-06	rs11893432	*	4.01E-11	58	0.581	rs16833197	*	**0.0195**
**FCGR2C**	1	26	3.00E-06	rs4554699	**	2.72E-08	24	**0.0158**	rs17411858	**	**0.00497**
**NIPSNAP3B**	9	3	4.00E-06	rs3780540	*	7.79E-04	4	0.551	rs3780540	*	0.31
**SCT**	11	34	4.00E-06	rs4963128	*	6.72E-05	52	0.691	rs10902178	*	**0.0456**
SERBP1	1	48	6.00E-06	rs3790569	*	5.61E-05	61	0.855	rs11807749	**	0.0682
PINX1	8	200	8.00E-06	rs17152571	*	1.46E-05	210	**0.0283**	rs17152345	*	**6.61E-04**
EXOC2	6	15	8.00E-06	rs908026	**	2.79E-05	21	0.382	rs7761186	*	**0.0396**

*Intronic SNP

**intergenic SNP

One gene out of four significant at this level in Whites, namely *FCGR2C*, was also nominally significant in African Americans. *PINX1*, which was only borderline significant in Whites, also showed a nominal significance in African Americans. Additionally, nominally significant SNPs where observed in *STAT1* and *SCT* genes and in the borderline significant *EXOC2* in the analysis of African Americans (Table 2), even though these genes did not reach significance in the gene-level analysis in that population.

The top genes identified for clinical phenotypes and autoantibody subsets are shown in S1–S4 Tables, in the left portion for the gene-level analyses based on ImmunoChip. *FCGR2C*, *STAT1*, and *FCGR3B* were significant in lcSSc, although *FCGR2C* and *FCGR3B* shared the most significant SNP rs455499 and the gene-level p-value for *FCGR2C* was more significant (S1 Table). In dcSSc, three genes (*IL34*, *ABBA*-1, and *VAC14*) were identified as significant, although they shared the most significant SNP rs11640251 and *IL34* had the best p-value in the gene-level analysis (S2 Table). In ACA+ patients, in addition to *STAT4*, six genes (*FCGR2C*, *SRCAP*, *PHKG2*, *LOC90835*, *RNF40*, and *FCGR3B*) reached significance in the ImmunoChip-based gene-level analysis, but the middle four genes shared the most significant SNP rs7188927 and *SRCAP* showed the best gene-level p-value (S3 Table), and *FCGR2C* and *FCGR3B* also shared the most significant SNP rs455499. Of these two genes, *FCGR2C* showed a more significant gene-level p-value like in the case of the lcSSc phenotype. Among ATA+ patients, in addition to *TNPO3*, *C16orf68*, *P2RX1*, *C3orf25*, *IFT122*, and *MBD4* reached significance in the gene-level analysis, with the last three genes sharing the same most significant SNP rs2307293 (S4 Table).

In the reverse approach we selected top non-HLA genes most significant in African Americans (p<10^−3^) based on the gene level analysis (Table 3; 13 genes but only 9 independent regions, due to the gene overlap).

**Table 3:** Genes most significant (at p<10^−3^) in African Americans in the gene-level analysis and the results for these genes in Whites, based on ImmunoChip.

			African	Americans				Whites			
Gene	Chr	N_SNPs_	P-value	Top SNP		Top SNP p-value	N_SNPs_	P-value	Top SNP		Top SNP p-value
*SCN4B*	11	4	1.20E-05	rs868344	^ǁ^	4.04E-03	4	0.544	rs671111	^§^	0.291
*SCN2B*	11	4	1.50E-05	rs868344	^§^	4.04E-03	3	0.687	rs671111	^§^	0.291
*MRPL28*	16	1	6.20E-05	rs743961	^‡^	6.72E-05	1	0.0863	rs3848368	**	0.0856
*TMEM8*	16	1	6.20E-05	rs743961	*	6.72E-05	1	0.105	rs3848368	**	0.0856
*NME4*	16	1	6.90E-05	rs743961	^‡^	6.72E-05	1	0.0869	rs3848368	**	0.0856
*DECR2*	16	1	7.50E-05	rs743961	^‡^	6.72E-05	1	0.0836	rs3848368	**	0.0856
*UNC5C*	4	8	1.22E-04	rs7697199	*	6.20E-05	6	**5.57E-04**	rs17381177	*	**7.84E-04**
*RAB11FIP3*	16	2	2.96E-04	rs743961	^‡^	6.72E-05	1	0.358	rs3785301	*	0.338
*PLCG2*	16	5	4.97E-04	rs4325546	*	3.15E-03	8	0.601	rs3936112	*	0.0874
*CLEC16A*	16	329	5.29E-04	rs1646066	**	5.39E-04	355	**0.0463**	rs16957854	*	**0.00169**
*ABCB4*	7	24	5.77E-04	rs17149601	*	2.16E-04	17	0.271	rs17149512	^ǁ^	0.083
*TCERG1*	5	1	7.61E-04	rs10056189	**	7.31E-04	1	0.135	rs10056189	**	0.171
*PHF19*	9	74	8.09E-04	rs1008382	*	1.32E-04	61	0.721	rs388040	*	**0.0426**

^ǁ^ 3’UTR SNP

^§^3’ downstream

^‡^5’ upstream

*intronic SNP

**intergenic SNP

Of these genes, only *UNC5C* (p = 5.57x10^-4^) and *CLEC16A* (p = 0.0463) were nominally significant in the gene-level analysis in Whites, and both these genes and *PHF19* harbored a nominally significant SNP in Whites (Table 3).

Among the 63 top genes (27 independent regions) selected based on SNP level analysis in African Americans (S5 Table; genes with best SNP p-value<10^−3^ in African Americans), 16 genes (12 independent regions) were also nominally significant in Whites at the gene level and 42 genes harbored at least nominally significant SNPs in Whites (25 different SNPs due to assignment of some SNPs to several genes at once). Since under the null hypothesis the expected number of nominally significant SNPs in Whites is ~1 (27x0.05 = 1.3), the results suggest some overlap in genetic susceptibility loci for SSc between Whites and African Americans.

### GWAS data analysis

We performed similar analyses also based on the GWAS genotyping which was, however, performed on different platforms in Whites and African Americans, and this made the results less comparable. The results are presented in S6–S11 Tables. In brief, among the genes previously identified in Whites, in addition to *TNFAIP3*, *ATG5* showed a nominal significance in the gene-level analysis in African Americans, potentially due to a denser coverage of this gene (81 vs 62 SNPs) on the 2.5 M Omni platform than on ImmunoChip (S6 Table).

Beyond the genes previously established as associated with SSc in Whites, only one gene, *TMEM175*, showed a borderline significant association in the gene-level analysis in Whites (p = 3.0x10^-6^) in GWAS. Its most significant SNP rs2290405 was shared with two other genes (*SLC26A1* and *DGKQ;* S7 Table). The analysis of the corresponding genes in the GWAS data in African Americans did not detect gene-level significance for these genes but *TMEM175* harbored a nominally significant SNP rs11946340 unlike the other two neighboring genes.

In the analysis of clinical phenotypes and autoantibody subsets, out of already established SSc susceptibility genes, *IRF5*, *TNPO3*, and *IRF8* were significant in lcSSc patients. There were also seven newly identified genes, of which four (*DGKQ*, *IDUA*, *TMEM175*, and *SLC26A1*) shared the same most significant SNP rs11724804; of these, *DGKQ* showed the best gene-level p-value. *IRF4*, *CCDC104*, and *TLR10* were also significant. Notably all these genes were at least nominally significant in the ImmunoChip-based gene-level analysis, except for *CCDC104* which is not on ImmunoChip (S1 Table). In dcSSc, in addition to *IRF5* and *TNPO3*, *CPSF4* and *ATP5J2* showed significant p-values in the gene-level analysis; they shared the same most significant SNP rs10235235, and *CPSF4* was more significant in the gene-level analysis. Except for *IRF5* and *TNPO3*, no gene reached statistical significance in the ACA+ subset in the GWAS-based gene-level analysis. In the ATA+ subset, *TLR10* and *TLR1*, sharing the same most significant SNP rs10024216, were significant (both reached only nominal significance in the ImmunoChip-based gene-level analysis) (S4 Table).

In the reverse analysis, considering the top genes identified in the gene-level analysis in African Americans (12 non-HLA genes but 11 regions due to the gene overlap, S8 Table), none of the corresponding genes was even nominally significant in Whites but 6 genes harbored nominally significant SNPs. Among the genes identified in the GWAS SNP-level analysis on African Americans as harboring most significant SNPs (p<10^−3^; 488 such genes but only 308 independent regions because of the gene overlap), 311 genes (255 independent regions) also harbored at least nominally significant SNPs in Whites. Eight genes (*SLC2A13*, *NRG3*, *SLC10A7*, *MKL1*, *DZIP1L*, *C8orf58*, *KIAA1967*, and *HDAC1;* seven independent regions, *C8orf58* and *KIAA1967* representing the same region) were nominally significant in both Whites and African Americans in the gene-level analysis. The results are presented in S9 Table (a). Five SNPs—rs2994241 in *C10orf27*/*ADAMTS14*, rs6025407 in *BMP7*, rs6796265 in *OSBPL10*, rs7734699 in *MRPS27*, and rs6075784 in *STK35* –were nominally significant in both Whites and African Americans. These five SNPs are marked in green in S9 Table (a), and their risk effects are shown in S9 Table (b).

We also catalogued genes identified in African Americans either in GWAS or ImmunoChip gene-level analysis (S10 Table), or by the top SNP p-value (with p<10^−3^) (S11 Table). In the few cases where the same SNP was top in both GWAS and ImmunoChip analyses, a slight p-value variation is explained by the QC procedures that eliminated different number of individuals from the ImmunoChip versus GWAS analysis.

Genes for SSc and other autoimmune diseases are enriched by the immune response genes [19–21]. One can expect, therefore, that SSc genes will be often involved in direct interactions. We used PathwayStudio to build an interaction network of known as well as 6 novel candidate genes (both significant and suggestive) (Table 3) identified by the gene-level analysis. Such networks may be useful by providing guidance to explore biological mechanisms underlying SSc risk. We found that two suggestive candidates, namely *EXOC2* and *PINX1*, interact with known genes associated with risk of SSc. The EXOC2 protein has been shown to bind LST1 [22], and PINX1 and STAT1 show protein/protein interaction [23] (S1 Fig).

## Discussion

We identified 4 novel candidate genes (*STAT1*, *FCGR2C*, *NIPSNAP3B*, and *SCT*) significantly associated and 4 genes (*SERBP1*, *PINX1*, *TMEM175* and *EXOC2*) suggestively associated with SSc in a gene level analysis in Whites. Some of these genes have been shown to be directly involved in immune response. For example, *FCGR2C* encodes a member of low-affinity immunoglobulin gamma Fc receptors. FCGR2C is found on the surface of many immune response cells. The gene encodes a transmembrane glycoprotein involved in phagocytosis and clearing of immune complexes. A suggestive novel gene, *SERBP1*, encodes a B-cell antigen, shown to predict anti-tumor immune response [24]. Another suggestive gene, *EXOC2*, is associated with innate immunity and has been shown to play a role in susceptibility to Crohn’s disease [25]. We note that the suggestive signal at *EXOC2* overlaps with the signal at *IRF4* previously described in the cross-disease meta-GWAS of SSc and rheumatoid arthritis [26], which points at the importance of this region in autoimmune conditions. This gene harbored a SNP rs908026 with a relatively strong statistical evidence for risk association (P = 2.8x10^-5^). Risk-associated SNPs were observed in other gene-level candidates as well. For example, rs11893432 (*STAT1* gene; p = 4.01x10^-11^) and rs4554699 (*FCGR2C* gene; p = 2.7x10^-8^) were significant at the GWAS level. The most significant SNPs in other novel candidates were: rs2290405 in *TMEM175* (p = 1.82x10^-6^), rs17152571 in *PINX1* gene (P = 1.5x10^-5^), rs3790569 in *SERBP1* gene (P = 5.6x10^-5^), rs4963128 in *SCT* gene (P = 6.7x10^-5^), and rs3780540 for *NIPSNAP3B* gene (P = 7.8x10^-5^). We admit that a further revalidation in an independent study of SSc in Whites is necessary.

Out of the 19 genes that were previously identified as harboring SSc susceptibility SNPs in Whites, only *TNFAIP3* was nominally significant in the gene-level analysis in African Americans. Previously, we showed that SNPs of *TNFAIP3* had a strong association with expression of matrix metalloproteinase 1 and 3 in fibroblasts of ethnically diverse patients in response to silica particle stimulation [27].

Several factors could have contributed to the absence of an association in African Americans for genes found in Whites. First, the distribution of clinical phenotypes is markedly different in the two populations, with a considerably higher proportion of the diffuse phenotype among African Americans (69%) as compared to Whites (31%). Our previous publication [6] shows differences in the genetic architecture of SSc clinical phenotypes. Thus clinical phenotype-specific analyses by ethnic group would be most meaningful, because they would allow for more accurate racial comparisons. Unfortunately, the limited number of African American participants precluded the phenotype or autoantibody subset analyses in the current study.

Second, the power of the analysis in the African Americans was limited because of the sample size. Moreover, the power of the analysis depends not solely on the sample size but also on the risk allele frequency. S9 Table (b) exemplifies that there is a considerable variation in the allele frequencies between the two populations, which could have contributed to the inter-ethnic differences. Third, even if the effects of *causal* SNPs are similar across ethnicities, GWAS-identified *tagging* SNP alleles can be in the opposite linkage phases in two given ethnic groups. This will result in the opposite effects of the tagging SNPs identified as significant in both African Americans and Whites, and the data in S9 Table (b) suggest exactly that: some SNPs identified as nominally significant have very similar frequencies but the opposite direction of the effect in African Americans and Whites. It is also possible that the causal SNPs are different in different ethnicities although the susceptibility genes are the same. A gene-level analysis of dense genotyping data, such as ours, should be able to capture the susceptibility genes even in case of the ethnic heterogeneity for causal alleles, unless a given ethnicity lacks causal variants in a potential susceptibility gene, in which case the gene will not be associated with disease in that ethnic group. The genes listed in Table 2, except for *NIPSNAP3B*, are densely SNP-genotyped in both Whites and African Americans. Thus it is not very likely that an individual SNP being poly- versus monomorphic has led to the loss of an association. For *NIPSNAP3B*, the top SNP in both populations was the same, rs3780540, and the MAF was actually higher in African Americans (0.116) than in Whites (0.0179), yet it was only significant in Whites.

An interaction network built using Pathway Studio detected a large number of interactions between genes associated with SSc risk. Genes with the largest number of interactions include *ITGAM*, *AIF1*, *STAT1*, *IL12RB2;* these genes form hubs of the network and are likely to be master genes in biological control of SSc risk. Two suggestive candidates, *EXOC2* and *PINX1*, are also part of this network.

As mentioned before, limitations of this study are (1) a relatively small sample size for African Americans, which prevents us from drawing any definite conclusion concerning the hitherto unresolved issue whether the same genes/SNPs influence SSc risk in different ethnic groups, and (2) the absence of independent validation cohorts. We acknowledge, therefore, that our analyses should be considered exploratory. Nevertheless, we carried out such analyses because the data are unique and their analysis may be important for the understanding of the role of ethnicity in the genetic architecture of SSc.

The results of our exploratory analysis might suggest that there exist both trans-racial and race-specific susceptibility loci for SSc, but further validation by independent studies, in particular a properly powered SSc GWAS in African Americans that allows subset analyses, is necessary to answer this question.

## Conclusions

A gene-level analysis focusing on the data generated by the ImmunoChip platform was performed on White and African American SSc patients. This study represents the first report of genetic analysis of African Americans with SSc. The gene-level analysis identified four novel candidate genes (*STAT1*, *FCGR2C*, *NIPSNAP3B*, and *SCT*) significantly associated with SSc in Whites. As an exploratory analysis we compared the results in Whites with those generated from African Americans. There was weak evidence of existence of SSc susceptibility loci that showed effects in both Whites and African Americans. Our findings need to be validated by independent studies, particularly due to the limited sample size of African Americans. The clinical phenotype and autoantibody subset analyses for Whites are also presented, but future studies should compare the phenotype- and autoantibody-stratified analyses in Whites and African Americans.

## Supporting information

S1 Table A catalogue of genes identified for limited SSc in Whites as harboring SNPs at p<10^−5^ in either ImmunoChip or GWAS data. (XLSX)

S2 Table A catalogue of genes identified for diffuse SSc in Whites as harboring SNPs at p<10^−5^ in either ImmunoChip or GWAS data. (XLSX)

S3 Table A catalogue of genes identified for ACA-positive SSc in Whites as harboring SNPs at p<10^−5^ in either ImmunoChip or GWAS data. (XLSX)

S4 Table A catalogue of genes identified for ATA-positive SSc in Whites as harboring SNPs at p<10^−5^ in either ImmunoChip or GWAS data. (XLSX)

S5 Table The top genes selected based on SNP level analysis (p<10^−3^) of ImmunoChip data in African Americans. (XLSX)

S6 Table The comparison of results of the gene-level analysis for Whites and African Americans for the genes previously shown as associated with SSc risk in Whites, based on GWAS data. (XLSX)

S7 Table Top novel genes identified by the gene-level analysis of GWAS data in Whites, with the comparative results from GWAS in African Americans. (XLSX)

S8 Table The top genes identified in the gene-level analysis of GWAS data in African Americans, with the comparative results in Whites. (XLSX)

S9 Table **(a)** The genes identified in the GWAS of African Americans as harboring most significant SNPs (p<10^−3^), with the genes at least nominally significant at the gene level in both African Americans and Whites shown at the top.**(b).** SNPs nominally significant in both African Americans and Whites.
(XLSX)

S10 Table A catalogue of genes identified in African Americans' gene-level analysis of either GWAS or ImmunoChip data. (XLSX)

S11 Table A catalogue of genes identified in African Americans as harboring SNPs at p<10^−3^ in either GWAS or ImmunoChip data. (XLSX)

S1 Fig The interaction network for known and novel candidate genes detected by the gene-level analysis. (DOCX)
