# Supplementary material for: Gene-level association analysis of systemic sclerosis: A comparison of African-Americans and White populations
Source: PLoS One. 2018 Jan 2;13(1):e0189498. doi: 10.1371/journal.pone.0189498 (PMC5749683; doi:10.1371/journal.pone.0189498)
Supplement: S1 Fig — (DOCX) [file pone.0189498.s012.docx]

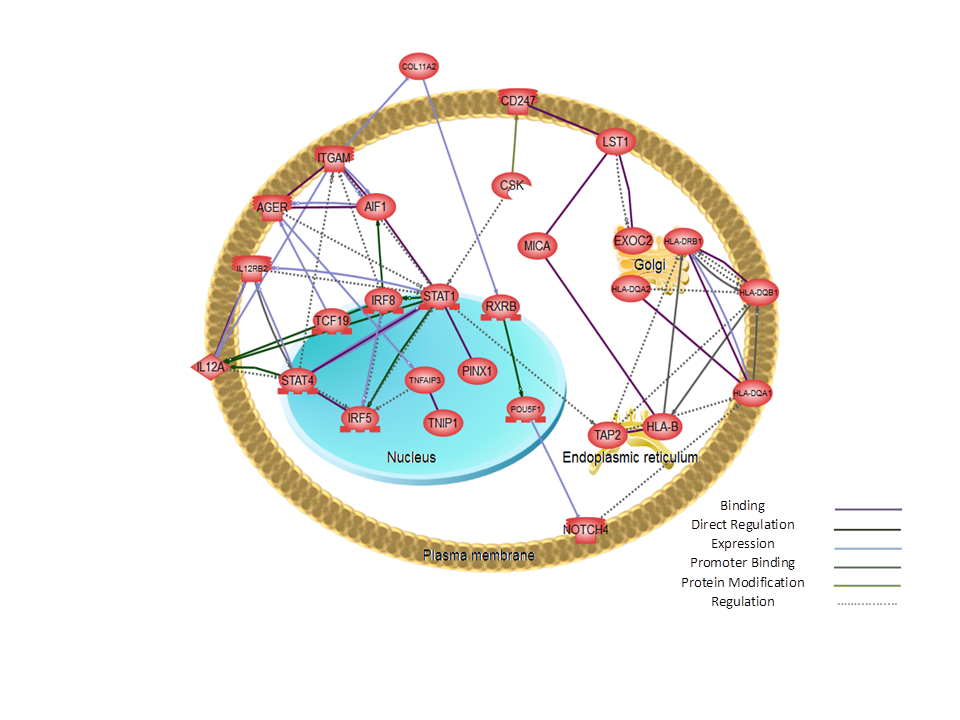


**S1 Fig**. The interaction network for known and novel candidate genes detected by the gene-level analysis.
